# Supplementary material for: A new large-bodied Pliocene seal with unusual cutting teeth
Source: R Soc Open Sci. 2020 Nov 11;7(11):201591. doi: 10.1098/rsos.201591 (PMC7735334; doi:10.1098/rsos.201591)
Supplement: A new large-bodied Pliocene seal with unusual cutting teeth [file rsos201591supp1.pdf]

A new large-bodied Pliocene seal with unusual cutting teeth

Supplemental Information

Contents

1. **Specimen List** **pg. 2–4**
2. **Figures** **pg. 5–8**
   Supplemental Figure 1: USNM PAL 475486 reconstruction.
   Supplemental Figure 2: USNM PAL 475486 reconstruction.
   Supplemental Figure 3: USNM PAL 534034 cranial fragments.
   Supplemental Figure 4: USNM PAL 534034 cervical vertebra
3. **Body Length Estimation** **pg. 8–11**
   Supplemental Table 1: Body length estimates for skull material.
   Supplemental Table 2: Body length estimates for humerus.
   Supplemental Table 3: Body length estimates for fossil seals.
   Supplemental Table 4: Total body length data.
   Supplemental Figure 5: USNM PAL 214909 *Monotherium*? *wymani.*
4. **Phylogenetic Characters** **pg. 11–21**
5. **Character Coding** **pg. 22–24**
   Supplemental Table 5:
6. **Parsimony Analysis Data** **pg. 25–28**
   Supplemental Table 6: Synapomorphies for clades.
   Supplemental Figure 6: Consensus tree for the equal weights parsimony with tip and node labels.
   Supplemental Table 7: Tip labels for Supplemental Figure 4.
   Supplemental Table 8: Node ages.
   Supplemental Table 9: Stratigraphic range data for taxa.
7. **References** **pg. 29–30**

1. Specimen List

Species, and relevant specimens assigned to them, used in this study for comparative purposes and coding of the morphological phylogenetic character matrix are listed below. For USNM specimens from the Paleobiology collection, EZIDs are included when available.

Enaliarctos emlongi: Holotype USNM PAL 250345.

Allodesmus kernensis: LACM 4320, LACM 9723.

Devinophoca claytoni: USNM PAL 553687 (cast of holotype Z14523).

"*Leptophoca proxima*": CMM-V-2021.

Sarcodectes magnus: USNM PAL 475486 (EZID: http://n2t.net/ark:/65665/3f15136ff-f662-4619-9b1c-7b0222daf511), USNM PAL 534034 (EZID: http://n2t.net/ark:/65665/31eb3c4a6-350e-4f4a-8f97-44950a11639f), USNM PAL 181601 (EZID: http://n2t.net/ark:/65665/350b4781d-5a43-4169-95b8-05f5998dc6fd).

"*Callophoca obscura*": USNM V 10434 (cast of lectotype IRSNB 1198-M203), IRSNB 1198-M203.

"*Mesotaria ambigua*": IRSNB 1156-M177.

"*Auroraphoca atlantica*": Holotype USNM PAL 181419 (EZID: http://n2t.net/ark:/65665/36c30cf22-a0c5-4015-98d6-6ecc6aebb885), USNM PAL 250290 (EZID: http://n2t.net/ark:/65665/35b133883-c9ac-49bb-846a-bcc8613d556c).

Monachinae gen. sp.: USNM PAL 186944, IRSNB 301, USNM PAL 263656, IRSNB 1116-M188.

"*Virginiaphoca magurai*": Holotype USNM PAL 639750.

"*Gryphoca similis*": USNM PAL 263625 (EZID: http://n2t.net/ark:/65665/35d6e3bc8-7765-41d4-96c3-f5f436c098fd).

Homiphoca sp.: USNM PAL 187228 (EZID: http://n2t.net/ark:/65665/395db7865-b359-41f3-96cf-4c0a0071bb2a).

Homiphoca capensis: MNHN.F.AFS 13, MNHN.F.AFS 31, MNHN.F.AFS 34, MNHN.F.AFS 35, MNHN.F.AFS 36, MNHN.F.AFS 37, MNHN.F.AFS 38, MNHN.F.AFS 39, cast SAM-PQ-L30080, cast SAM-PQ-L15441, cast SAM-PQ-L15652, SAM-PQ-L4638, SAM-PQ-L12869, SAM-PQ-L2161, SAM-PQ-L15849A, SAM-PQ-L30118, SAM-PQ-L30424, SAM-PQ-L10130, SAM-PQ-L10996.

Acrophoca longirostris: Holotype MNHN.F.SAS 563, MNHN.F.SAS 1654, MNHN.F.SAS 731, MNHN.F.SAS 648, MNHN.F.SAS 573, USNM PAL 421632, MNHN.F.PPI 274, MNHN.F.PPI 273.

Hadrokirus martini: Holotype MNHN.F.SAS 16276.

Piscophoca pacifica: Holotype MNHN.F.SAS 564, MNHN.F.SAS 501, MNHN.F.SAS 72, MNHN.F.SAS 682, MNHN.F.SAS 488, MNHN.F.SAS 678.

Australophoca changorum: Holotype USNM PAL 438707, USNM PAL 438712.

Monotherium? wymani: USNM PAL 214909 (cast of holotype MCZ 8741).

Beaumaris monachine: NMV P232849

Arctocephalus sp.: NMV C1987, NMV C38182, NMV C33579.

Odobenus rosmarus: NHMUK 331.h. NHMUK 331.c, USNM 396932, USNM 22014, USNM 199528.

Erignathus barbatus: NHMUK 1887.9.28.1, NHMUK 1938.11.26.1, NHMUK 1937.10.23.9, USNM 16116, USNM 230950, USNM 7107.

Cystrophora cristata: NHMUK 1956.11.7.1, NHMUK 1844.2.2.81, USNM 550317, USNM 550317.

Halichoerus grypus: NHMUK 1962.3.6.1, NHMUK 1951.11.28.1, USNM 446405, USNM 504480, USNM 594208.

Histriophoca fasciata: NHMUK 1966.12.7.2, NHMUK 1965.7.19.8, USNM 59397, USNM 16484.

Pagophilus groenlandicus: NHMUK 1938.12.10.1, NHMUK 1946.6.18.3, USNM 59397, USNM 3517, USNM 3505.

Pusa sibirica: NHMUK 1965.9.6.1, USNM 175689, USNM 550038, USNM 550028.

Pusa hispida: NHMUK 1997.585, NHMUK 1938.12.10.5, USNM 16106, USNM 49472, USNM 225779, USNM 225786.

Pusa caspica: NHMUK 1965.7.19.2, USNM 341615, USNM 341616, USNM 341617.

Phoca largha: NHMUK 1891.12.18.7, NHMUK 1873.11.5.7.

Phoca vituline: NHMUK 329.i, USNM 15276, USNM 63018, USNM 4713.

Monachus monachus: NHMUK 1894.7.27.2, NHMUK 1951.4.17.1, NHMUK 1892.11.7.1, NHMUK 1894.7.27.1, USNM 219059, USNM 23250.

Neomonachus tropicalis: NHMUK 1889.11.5.1, USNM 22543, USNM 446408, USNM 20994, USNM 102527.

Neomonachus schauinslandi: USNM 504893, USNM 243839, USNM 396001, USNM 395999, USNM 243839, USNM 395997, USNM 181250, USNM 396001, USNM 395988.

Mirounga leonina: NMV C25797, NMV C7391, NMV C7414, USNM 484893, USNM 484880.

Mirouna angustirostris: USNM 15270, USNM 12441, USNM 21891, USNM 260867.

Hydrurga leptonyx: NMV C23613, NMV C36966, NMV C36591, NHMUK 1959.12.17.4, USNM 396931, USNM 104557.

Lobodon carcinophaga: NMV C30845.1, NMV C25039, NHMUK 1908.2.20.51, USNM 504741, USNM 310693, USNM 550078, USNM 550077.

Leptonychotes weddellii: NHMUK 1951.8.28.17, NHMUK 1908.2.20.26, NHMUK 1951.8.28.16, USNM 550118, USNM 550074, USNM 484887.

Ommatophoca rossii: NHMUK 1965.8.2.1, NHMUK 1965.12.20.1, NHMUK 1949.2.3.1, USNM 275206, USNM 302975, USNM 270316, USNM 270321.

Acinonyx jubatus: MU-IMP 501.

Lycaon pictus: SMNS 4461.

For all other species, comparisons were made, and coding was done using the literature.

2. Figures

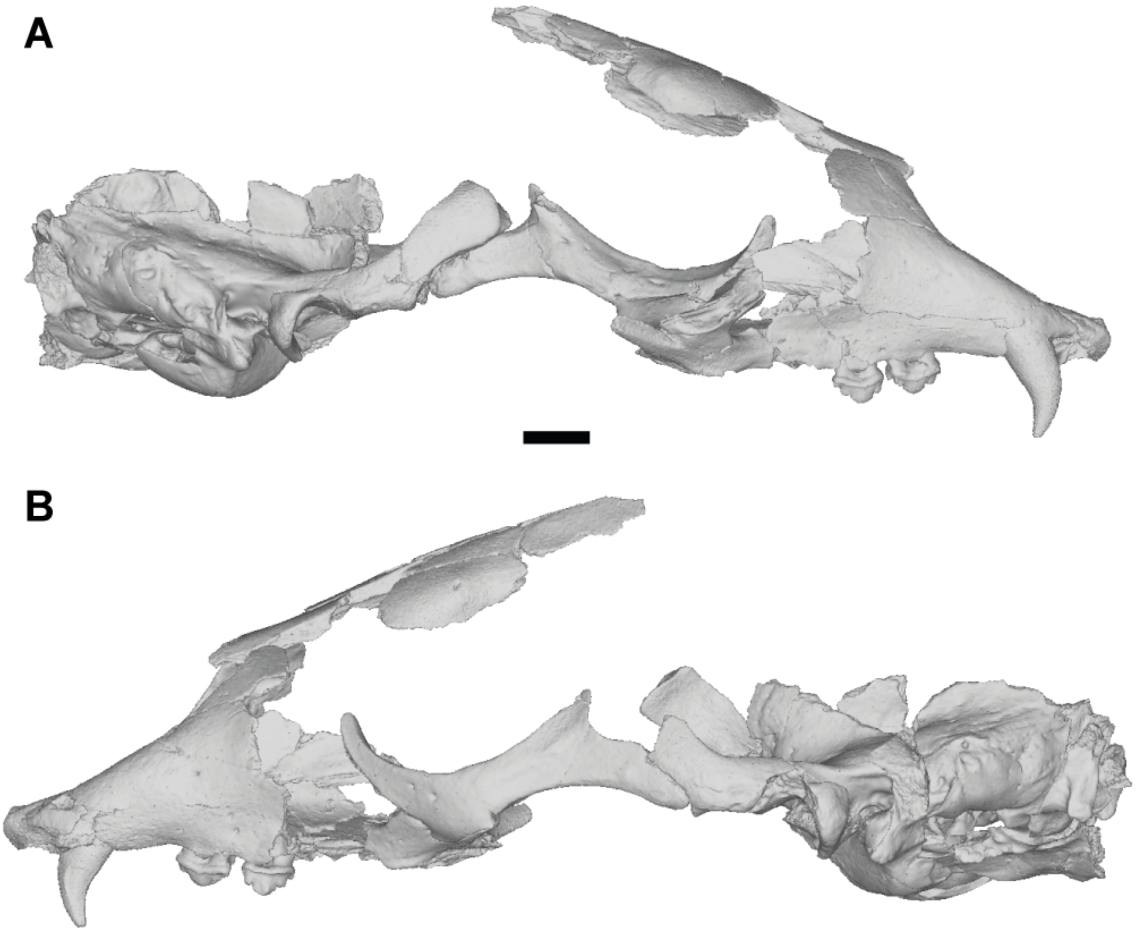

Supplemental Figure 1. Reconstructed crania USNM PAL 475486. Surface scan of the reconstructed holotype in right (A) and left (B) lateral views. Scale bar 2 cm.

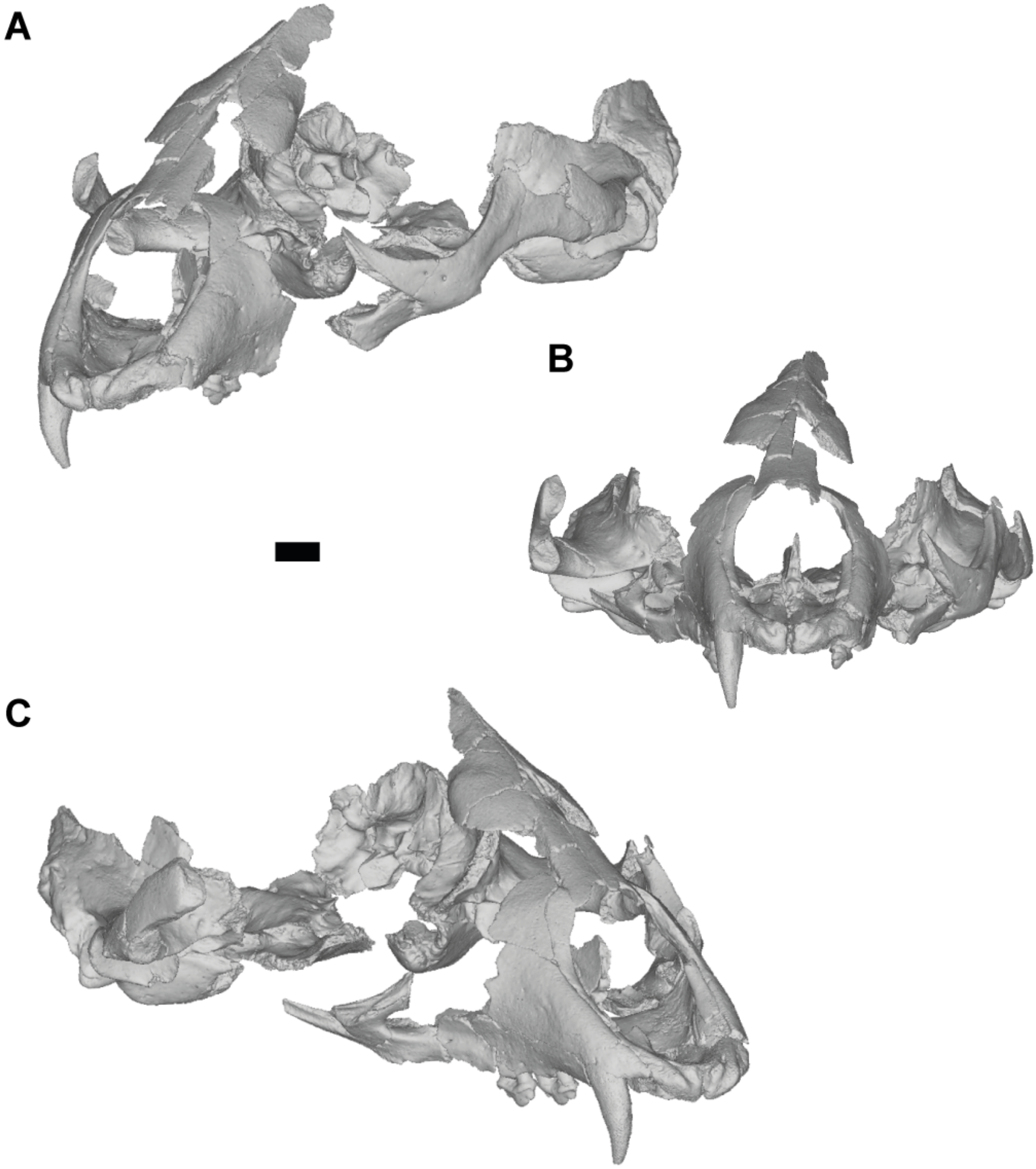

Supplemental Figure 2. Reconstructed crania USNM PAL 475486. Surface scan of the reconstructed holotype in left oblique (A), anterior (B), and right oblique (C) views. Scale bar 2 cm.

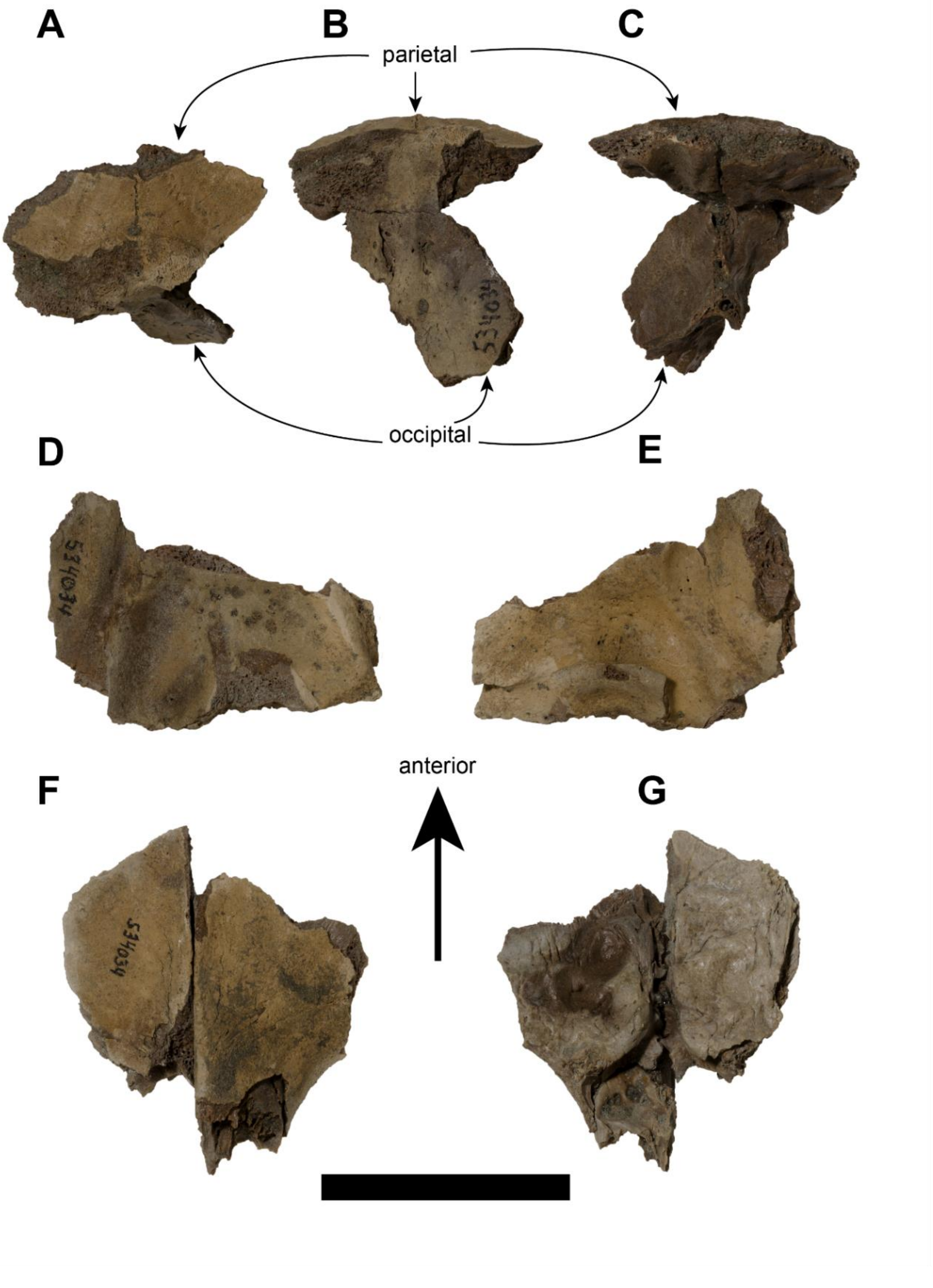

Supplemental Figure 3. Cranial fragments of USNM PAL 534034. Cranial fragments, including parietal-occipital (A, B, C), basicranial fragment (D, E), and frontal (F, G). (A) parietal-occipital in dorsal view. (B) parietal-occipital in posterior view. (C) parietal-occipital in anterior view. (D) basicranial fragment in ventral view. (E) basicranial fragment in dorsal view. (F) frontal fragment in dorsal view. (G) frontal fragment in ventral view. Scale bar 5 cm.

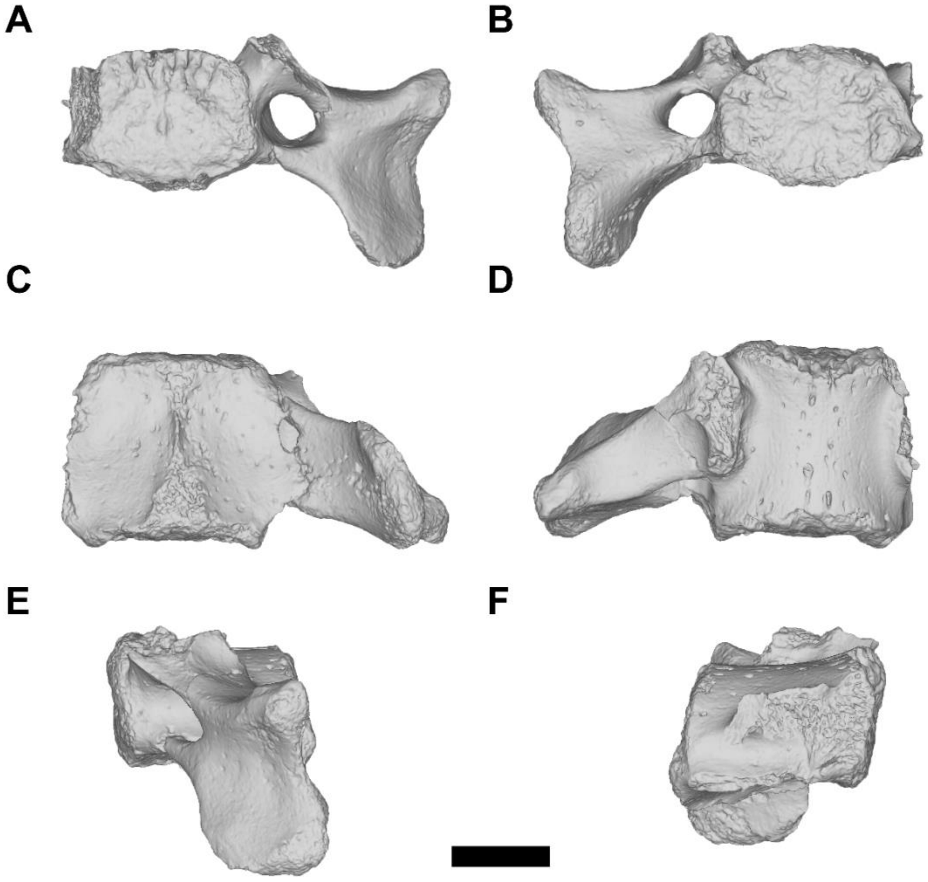

Supplemental Figure 4. Cervical vertebra of USNM PAL 534034. (A) anterior view. (B) posterior view. (C) ventral view. (D) dorsal ventral view. (E) left lateral view. (F) right lateral view. Scale bar = 2 cm.

3. Body Length Estimation

Supplemental Tables 1 and 2 outline details behind the body mass estimations of the holotypes and paratypes, as presented in the manuscript. Supplemental Tables 3 and 4 indicate total length estimates and metrics for Figure 17 and Figure 18 in the main text.

Supplemental Table 1. Body length estimations from skull measurements, using the predictive regression equations of [2].

Specimen Number	Equation	Metric (mm)	Regression equation value	Body length estimate (m)
USNM PAL 475486	LUTR	109.76	3.54	3.47
	LUPC	91.32	3.52	3.33
USNM PAL 181601	WB	53.91	3.45	2.82
	LB	51.42	3.19	1.56

Supplemental Table 2. Body length estimation from the total length (180.44 mm) of the humerus of USNM PAL 534034, using ratios reported in the literature.

Taxon	Reference	Humerus/Body length ratio	Body length estimate (m)
Ommatophoca rossi	[3]	0.0595	3.03
Leptonychotes weddelli	[4]	0.065	2.78

Supplemental Table 3. Body length estimation for other taxa, using various methods [2-5]. Measurements for USNM PAL 181419 and USNM PAL 639750 taken from Dewaele *et al*. [6]. Measurements for *Pliophoca etrusca* taken from Berta *et al*. [7]. Measurement for "*Gryphoca similis*" taken from Koretsky & Ray [1]. Measurement for USNM PAL 187228 taken from figures in Dewaele *et al*. [Figure 3, pg 8; 6] using Image J. Measurement for USNM PAL 214909 taken from Supplemental Figure 5 (below) using Image J. Measurements for *Hadrokirus martini* taken directly from the specimen.

Specimen Number	Taxon	Equation	Metric (mm)	Regression equation value	Body length estimate (m)	Mean estimate (m)
USNM PAL 214909	*Monotherium? wymani*	WB	41.40	3.33	2.12	1.64
		LB	34.50	3.06	1.16	
USNM PAL 187228	*Homiphoca* sp.	*O. rossii* hum/body length	131.30	-	2.21	2.12
		L. weddellii hum/body length		-	2.02	
USNM PAL 181419	"*Auroraphoca atlantica*"	*O. rossii* hum/body length	159.30	-	2.68	2.57
		L. weddellii hum/body length		-	2.45	
USNM PAL 639750	"*Virginiaphoca magurai*"	*O. rossii* hum/body length	134	-	2.25	2.16
		L. weddellii hum/body length		-	2.06	
MNHN.F.SAS 16276	*Hadrokirus martini*	All subsets	52.70 (CW),	3.43	2.70	2.70

		Total Length	56.90 (OCB)			
MSNUPI-13993	*Pliophoca etrusca*	CW	44.00	3.01	1.03	1.68
		O. rossii hum/body length	125.00	-	2.10	
		L. weddellii hum/body length		-	1.92	
USNM PAL 263625	"*Gryphoca similis*"	*Ph. vitulina* hum/body length	125.00	-	1.65	1.60
		P. sibirica hum/body length		-	1.54	

Supplemental Table 4. Body length for extant and extinct monachines, and extant phocines. Asterisk (*) indicates a body length estimation.

**Taxon**	**Body Length (m)**	**Reference**
Monachus monachus	2.80	[8]
Neomonachus schauinslandi	2.34	[8]
Neomonachus tropicalis	2.40	[8]
Mirounga leonina	5.00	[8]
Mirounga angustirostris	4.50	[8]
Lobodon carcinophaga	2.60	[8]
Ommatophoca rossii	3.00	[8]
Hydrurga leptonyx	3.80	[8]
Leptonychotes weddellii	2.60	[8]
Cystophora cristata	2.70	[8]
Halichoerus grypus	2.20	[8]
Pagophilus groenlandicus	1.60	[8]
Phoca vitulina	1.80	[8]
Pusa hispida	1.50	[8]
Australophoca changorum	0.68*	[9]
Piscophoca pacifica	1.96*	[10]
Acrophoca longirostris	1.91*	[10]
Homiphoca capensis	1.80*	[10]

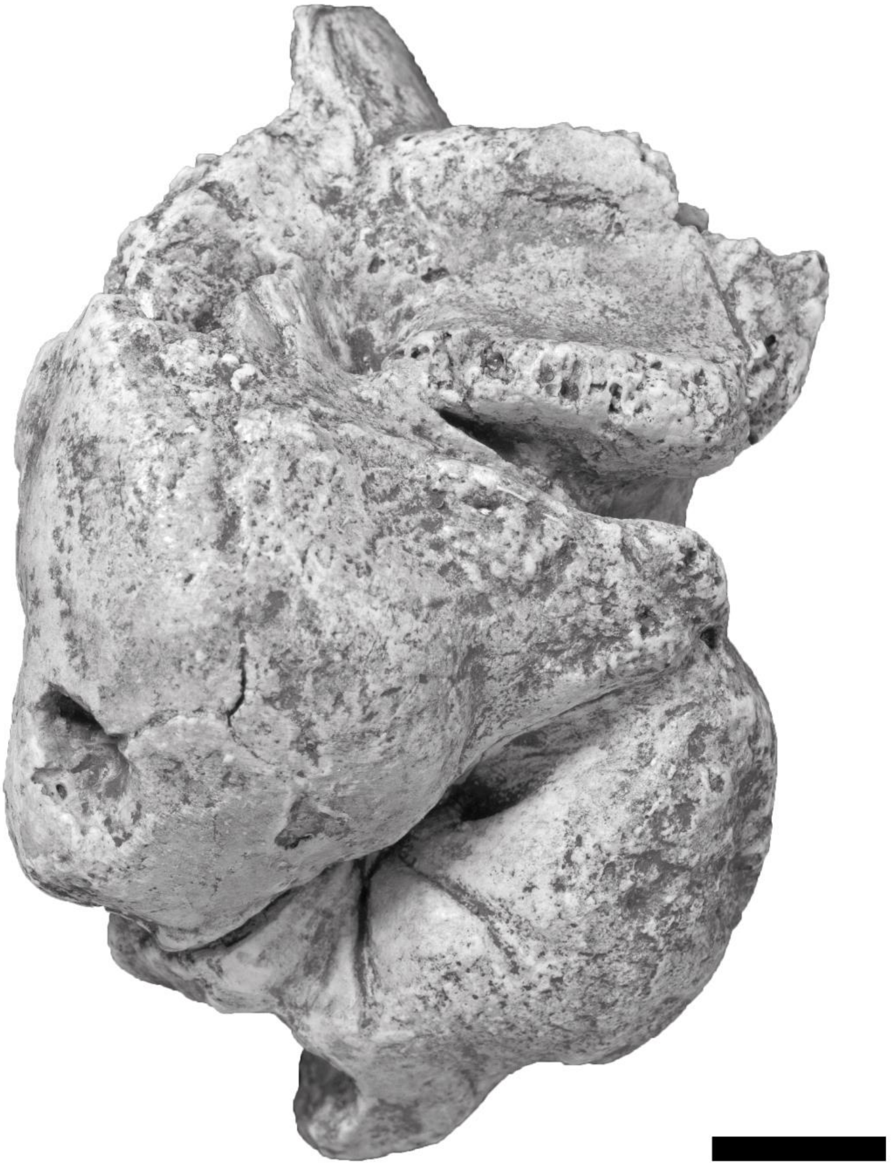

Supplemental Figure 5. USNM PAL 214909, cast of holotype (MCZ 8741) of *Monotherium? wymani*, in ventral view. Scale bar = 1 cm.

4. Phylogenetic Characters

Characters that are modified or sourced from other studies are stated as such in text.

1. Tooth rows: 0) parallel; 1) diverging posteriorly; Modified [11, 12].

2. Constriction of palate at level of P1 and P2: 0) absent; 1) present; [12].

3. Alveolar process of maxilla: 0) facing ventrally; 1) facing anteroventrally posterior to P1; [12].

4. Number of upper incisors: 0) six; 1) four; [12, 13].

5. Upper incisors: 0) in straight line; 1) in a curved line (slanted); [7, 14].

6. Upper incisors, shape in cross-section: 0) round; 1) laterally compressed; [7, 15, 16].

7. Transverse groove on at least the mesial incisor: 0) present; 1) absent; [11, 12].

8. Upper incisors, relative size: 0) lateral-most incisor relatively larger than others; 1) lateral-most incisor equal in size to remaining incisors; Modified [7, 15, 16].

9. Procumbent upper incisors: 0) absent; 1) present; [7, 16].

10. Anterior alveolar border of the upper incisors facing anteroventrally: 0) absent; 1) present; [12].

11. Diastema between P4 and M1 relative to other postcanine diastemas: 0) large; 1) similar size or reduced; Modified [7, 17].

12. Upper postcanine teeth, diastemas: 0) average length of diastemas between postcanines less than postcanine alveoli length (mesial-distal); 1) average length of diastemas between postcanines close to equal to or greater than alveoli length (mesial-distal) of postcanines; (calculated using postcanines anterior to diastema, i.e. not using the P5 alveoli).

13. P2, P3, P4 and M1: 0) at least one is triple rooted; 1) at least one is single-rooted; 2) all are double-rooted; Modified [11, 12].

14. M1: 0) equal in size to P4; 1) smaller than P4; [7].

15. M2: 0) present; 1) absent; [11, 12].

16. Elevation of the labial cingulum below the main cusp of postcanine teeth: 0) absent; 1) present; Modified [12].

17. Accessory cusps of postcanine teeth: 0) very small or weakly developed (longer than high); 1) well developed (higher than long); Modified [12, 18].

18. Postcanine teeth, main cusps (on uppers the paracone, on lowers the protoconid) and accessory cusps: 0) mesial and distal edges straight (or close to) forming triangular peaks; 1) mesial and distal edges curved, creating a globular cusp profile; (not coded for taxa lacking accessory cusps).

19. Postcanine teeth, notch condition (where the profile of the edges of the accessory cusps form a divet with the posterior edge of the main cusp): 0) absent; 1) present; (not coded for taxa lacking accessory cusps).

20. Postcanine/canine enamel surface: 0) smooth texture; 1) apicobasal ridges (crenulated texture) present.

21. Postcanine accessory cusps: 0) absent; 1) 1-2 ; 2) 3 or more; Modified [7, 14].

22. Distolingual projection of cingulum of upper premolars: 0) absent; 1) present; Modified [12, 13].

23. Lingual shelf (an occlusal-facing surface projecting lingually from the crown) on premolars: 0) absent; 1) present.

24. Crowns of postcanines: 0) labiolingually compressed; 1) labiolingually broad; [7, 19].

25. Premolars: 0) parallel to toothrow axis; 1) obliquely oriented in toothrow; [7, 14].

26. Upper and lower postcanine teeth, occluding: 0) no (interdigitating); 1) yes; Modified [7].

27. Lower postcanine teeth, lingual cingulum shelf: 0) absent; 1) present; 2) present posteriorly only.

28. Mandibular condyle: 0) at or slightly above level of toothrow; 1) well elevated above toothrow [7, 11].

29. Coronoid process and mandibular condyle of mandible: 0) coronoid process extends well dorsal from the mandibular condyle, posterior profile of ramus open (not a tight semicircle); 1) coronoid process extends well dorsal from the mandibular condyle, posterior profile of ramus closed (a tight semicircle); 2) coronoid process does not extend well dorsal from the mandibular condyle, posterior profile of ramus reduced.

30. Angular process of dentary: 0) large process protruding medially and posteriorly; 1) more a knob than a process and weakly protruding or not protruding medially; [12].

31. Shape of body of mandible in posterior view: 0) sigmoid; 1) sub-rectilinear [12].

32. Relative position of lower incisors: 0) mesial incisor posterior to lateral incisor; 1) mesial incisor level or slightly anterior to lateral incisor; 2) mesial and lateral incisors orientated antero-posteriorly; (Not coded in taxa with only one lower incisor) Modified [12].

33. Shape of alveolus of lower canine: 0) circular to subcircular; 1) very elliptic; [12].

34. Mandibular symphysis: 0) terminates at p2; 1) terminates at p3; 2) terminates at p4; 3) terminates at p1; Modified [7, 14].

35. Masseteric fossa: 0) absent (indistinct) or shallow; 1) deep and well defined.

36. Post-dental ridge of mandible: 0) absent; 1) present; (coded for skeletally mature specimens only) Modified [20].

37. p4-m1 alveoli: 0) similar in size; 1) p4 alveolus smaller than m1 alveolus; 2) p4 alveolus larger than m1 alveolus; [7, 14].

38. Upper and lower postcanines: 0) relatively the same length-width ratio; 1) upper postcanines more robust (more labial-lingually wider) than lower postcanines; (not coded for non-crown-pinnipeds due to lack of homodonty).

39. Premaxilla and palatine fissure in ventral view: 0) premaxilla short, palatine fissure antero-posteriorly extended; 1) premaxilla short, palatine fissure reduced (small); 2) premaxilla elongated, palatine fissure posteriorly positioned.

40. Palatal groove (begins at a foramen on the posterior end of the maxilla: 0) absent, or foramen is present only; 1) palatal groove begins posterior to postcanines; 2) palatal groove begins at the level of the P5; 3) palatal groove begins at the level of the P4, or anterior to it.

41. Contact between premaxilla and nasal: 0) present; 1) absent; [12].

42. Premaxilla-maxilla suture: 0) lateral to nasal cavity; 1) at least partially included in nasal cavity; Modified [12, 13].

43. Opening of nasal cavity: 0) short and opening more anteriorly than dorsally; 1) long and opening more dorsally than anteriorly; Modified [12].

44. Lateral border of opening of nasal cavity in lateral view: 0) rectilinear or weakly concave; 1) strongly or dominately concave; Modified [12].

45. Fused nasals even in subadult specimens: 0) absent; 1) present; [12].

46. Position of posterior end of nasals: 0) anterior to maxilla-frontal suture; 1) posterior to maxilla-frontal suture but greatly anterior to the level of the jugal-squamosal suture; 2) almost reaches the level of the jugal-squamosal suture; Modified [11, 12].

47. Anterior end of frontals: 0) not inserted between nasals; 1) inserted medially between nasals; [11, 12].

48. Preorbital (antorbital) process: 0) angular and connected to the anterior margin of the orbit by an uninterrupted crest; 1) separated from the ventral border of the orbit; 2) Preorbital process absent; Modified [12].

49. Jugal in ventral view: 0) enlarged and flattened origin for masseter muscle, which does not interrupt the profile of the zygomatic arch; 1) enlarged and flattened origin for masseter muscle, which interrupts the anterior profile of the zygomatic arch with a medio-lateral bulge; 2) origin for masseter muscle indistinct.

50. Zygoamtic arch (ventral view), maxilla process: 0) maxillary process of zygomatic arch laterally directed, enlarging the ventral-anterior border of the zygomatic-interorbital area; 1) maxillary process of zygomatic thin and ventrally directed, narrowing the ventral-anterior border of the zygomatic-interorbital area; 2) maxillary process of zygomatic wide and ventrally directed, narrowing the ventral-anterior border of the zygomatic-interorbital area.

51. Maxillary process of jugal (at the level of the anteroventral border of the orbit) in lateral view: 0) thin and low and increasing progressively posteriorly; 1) thick and high and increasing abruptly posteriorly; [12].

52. Position of anterior opening of infraorbital foramen in ventral view: 0) anterior to M1; 1) level or posterior to M1; [12].

53. Nasolabialis fossa: 0) absent 1) present; [12].

54. Anterior end of jugal in dorsal view: 0) lateral to infraorbital foramen; 1) above or medial to the lateral margin of the infraorbital foramen; [12].

55. Ventral edge of the zygomatic arch, in anterior view: 0) dorsal to alveolar plane; 1) level with or ventral to the alveolar plane; Modified [12].

56. Mortised jugal-squamosal suture: 0) absent; 1) present; [11, 12].

57. Supraorbital process of frontal: 0) absent; 1) present; Modified [11, 12].

58. Least interorbital width: 0) occurs in posterior-most portion of interorbital bridge; 1) occurs in anterior half of interorbital bridge; [11, 12].

59. Least interorbital width/bizygomatic width ratio: 0) low (much lower than 0.3); 1) high (at least equal to 0.3); [12].

60. Post-auricular zygomatic shelf (horizontal to near horizontal shelf of bone): 0) absent; 1) present.

61. Squamosal portion of zygomatic in ventral view: 0) anterior extent of glenoid approaches jugal; 1) jugal separated from anterior extent of glenoid.

62. Superior surface of squamosal portion of zygomatic process, posterior to postorbital process, lateral view: 0) convex to flattened; 1) concave.

63. Major axes of glenoid fossae: 0) parallel to sub-parallel; 1) convergent posteriorly (lateral edge of posterior border glenoid fossa transversley level to medial border of anterior edge of glenoid fossa); Modified [12].

64. Anterior extension of the EAM as a deep groove on the lateral surface of the post-glenoid process: 0) absent; 1) present; (Coded for skeletally mature specimens only).

65. Basicranium (Basisphenoid and Basioccipital): 0) Straight to weakly transversley concave; 1) Vaulted (strongly transversley concave); Modified [15].

66. Anterior profile of the tympanic: 0) straight or slightly concave; 1) strongly concave (with the ectotympanic tubercle and anterior extent almost forming a right angle).

67. Orientation of medial margins of tympanic bullae: 0) diverging posteriorly; 1) parasagittal; 2) converging posteriorly; Modified [12].

68. Lateral end of tympanic bulla: 0) medial to level of mid-width of glenoid fossa; 1) lateral to level of mid-width of glenoid fossa; [12].

69. Ectotympanic tubercle: (ventral view) 0) medial or level to lateral surface of mastoid; 1) lateral to mastoid due to lateral elongation.

70. Fundus of EAM in lateral view: 0) entirely or partially visible; 1) completely obscured by the ectotympanic tubercle.

71. Inflation of tympanic bulla: 0) absent or weak ; 1) strong; Modified [12, 21].

72. Carotid foramen: 0) Contained within body of entotympanic bulla; 1) Located on a distinct medially projecting flange of the entotympanic. (Not coded when entotympanic is consisted only of carotid canal, and not bulbous, Otariidae).

73. Border of carotid foramen: 0) Tympanic separates carotid foramen from basioccipital; 1) Basioccipital forms medial border of carotid foramen; (only coded for specimens with tympanic still attached to the basicranium).

74. Carotid canal and jugular foramen: 0) carotid foramen runs the medial length of the tympanic, ending close to the jugular foramen; 1) carotid foramen separated from the jugular foramen by basicapsular fissure.

75. Tympanohyal fossa: 0) small and contained within an enlarged pit lateral to tympanic, containing the stylomastoid foramen; 1) present on the tympanic bulla, fossa is a shallow pit; 2) present on the tympanic bulla, fossa is deep, opening into distinct sulcus.

76. Mastoid, diagastric groove (leading to external cochlear foramen): 0) absent or indistinct; 1) present; 2) present, enlarged into a digastric pit.

77. Stylomastoid foramen and auricular foramen: 0) separated or nearly separated by apophysis arising from mastoid and/or tympanic bulla; 1) share an elongate arcuate opening; 2) are confluent with ovoid aperture.

78. Posterior opening of carotid canal: 0) full border visible in ventral view; 1) not visible in ventral view because of the strong inflation of the posterior part of the ectotympanic (only ventral aspect of border visible); Modified [11, 12].

79. Posterior opening of carotid canal and posterior lacerate foramen: 0) clearly separated; 1) coalescent; Modified [12, 16].

80. Posterior lacerate foramen: 0) small; 1) large and medial to tympanic bulla; [12, 22].

81. Orientation of pit for stylohyal: 0) ventral and/or posterior; 1) ventral and anterior; Modified [11, 12].

82. Posteromedial extension of the tympanic, past the carotid foramen: 0) absent, bulla contacts and is fused to the exoccipital and mastoid; 1) absent (bulla separated from the exoccipital and mastoid); 2) present, part of the profile and shape of the inflated bulla (extension medially convex in profile); 3) present, separate from the profile of the bulla (extension medially concave in profile); Modified [12].

83. Development of a lip of the anteromedial region of the pars mastoidea, which abuts the posteromedial extension of the tympanic dorsally and ventrally: 0) absent; 1) absent, but extension dorsally framed by mastoid; 2) present; Modified [12].

84. External cochlear foramen: 0) opening laterally off the tympanic bulla, not framed by the mastoid; 1) framed by the mastoid and tympanic bulla, opening ventrally; 2) framed by the mastoid and petrosal, opening ventrally ; (coded inapplicable for Lobodontins due to mastoid lip, and taxa without an inflated bulla).

85. Dome-like elevation of the uncovered part of the petrosal posterior to the posterior edge of the tympanic: 0) absent; 1) present; [12] (not coded for taxa with no exposed petrosal).

86. Petrosal, dorsal region: 0) unexpanded; 1) expanded; [7, 11].

87. Petrosal, dorsal region, subarcuate fossa (cerebellar fossa, floccular fossa): 0) wide open, ovoid outline; 1) narrow, almost slit-like; 2) wide open, ovoid outline in medial view only, due to dorsal covering of bone.

88. Petrosal apex: 0) relatively flat dorso-ventrally; 1) relatively swollen and bulbous dorso-ventrally.

89. Profile of petrosal apex: 0) angular; 1) rounded; 2) flattened; Modified [12, 15].

90. Relationship between facial nerve canal, fossa for vestibulocochlear nerve and roof of internal auditory meatus: 0) roof of internal auditory meatus present; 1) facial nerve canal and fossa for vestibulocochlear nerve separated; Modified [11, 12].

91. Shape of head of malleus: 0) broad and circular; 1) slender and elliptic; [12].

92. Petrosal and tympanic bulla: 0) tympanic bulla covers the petrosal posteriorly; 1) tympanic bulla does not cover the petrosal posteriorly, petrosal dorsal to the level of the basioccipital; 2) tympanic bulla does not cover the petrosal posteriorly, petrosal ventral to (and offsets the tympanic bulla) past the level of the basioccipital; Modified [7].

93. Heavily pachyosteosclerotic mastoid: 0) absent; 1) present; [7, 11].

94. Mastoid (lateral view): 0) mastoid process posterior to tympanic bulla; 1) mastoid process in line antero-posteriorly to tympanic bulla.

95. Dorso-lateral profile of the mastoid: 0) continuous, no enlarged digastric protuberance present; 1) digastric protuberance is enlarged and interrupts the profile of the mastoid; (coded for skeletally mature specimens only, not coded for non-phocids as they lack a pachyosteosclerotic mastoid).

96. Attachment for the sternomastoid muscle on the mastoid: 0) separate from the rest of the mastoid surface (on the lateral-ventral surface); 1) part of the continuous mastoid surface (not separate, and facing in the same orientation as the rest of the mastoid); (not coded for non-phocids as they lack a pachyosteosclerotic mastoid).

97. Mastoid visible in dorsal view: 0) no; 1) mastoid visible due to posterior extension; Modified [11, 12].

98. Relation of paroccipital process to mastoid: 0) paroccipital process connected to mastoid by a high and continuous ridge; 1) paroccipital process well separated from mastoid; [11, 12].

99. Direction of occipital condyles in occipital view: 0) vertical; 1) diverging dorsally; [12].

100. Occiput proportions: 0) ratio occiput height/mastoid width superior to 0.5; 1) ratio less than 0.5; [12].

101. Orientation of pterygoids: 0) vertical; 1) laterally tilted and flaring posteriorly; [12].

102. Pterygoid Hamulus: 0) present; 1) absent.

103. Postero-ventral extension of the vomer: 0) not extending to the posterior edge of the palatine; 1) extending to the posterior edge of the palatine.

104. Alisphenoid canal: 0) present; 1) absent; Modified [11, 12].

105. Ventral extension of bony tentorium: 0) weak (processus tentoricus absent); 1) approaches or reaches floor of braincase (processus tentoricus present); Modified [12, 23].

106. Atlas, transverse foramen: 0) full margin of foramen not visible in dorsal view; 1) full margin of foramen visible in dorsal view; Modified [12, 15].

107. Atlas, direction of transverse process in lateral view: 0) oblique; 1) sub-vertical; [12].

108. Atlas, deep fossa for m. rectus capitis dorsalis minor: 0) absent; 1) present; [12].

109. Cervical vertebrae 3-6, tubercle and lamina of transverse process: 0) fused or poorly isolated from each other; 1) clearly isolated from each other; [12].

110. Scapula, major orientation of posterior border in lateral view: 0) proximodistal (dorsal component greater than posterior component); 1) anteroposterior (posterior component greater than dorsal component); [12].

111. Scapula, acromion process: 0) knoblike; 1) reduced; [12, 15].

112. Scapula, scapular spine: 0) unreduced (i.e. the spine almost reaches the dorsal edge of the scapula); 1) reduced (i.e. the spine almost totally disappears and is reduced to its most proximal portion and the acromion); Modified [12, 22].

113. Scapula, scapular spine: 0) vertical or slightly tilted anteriorly; 1) tilted posteriorly; [12].

114. Scapula, supraspinous fossa subdivided: 0) absent; 1) present.

115. Scapula, supraspinous fossa: 0) smaller than infraspinous fossa; 1) same size or larger than infraspinous fossa; [11, 24].

116. Humerus, deltopectoral crest: 0) long (continuous and almost reaches the distal epiphysis); 1) short (stops abruptly approximately at mid-length of diaphysis); Modified [12, 15].

117. Humerus, deltopectoral crest: 0) anterior edge of humerus attenuates gradually, distally; 1) anterior edge of humerus terminates abruptly distally; Modified [24].

118. Humerus (lateral view), deltopectoral crest: 0) concave surface at origination of bracialis (lateral overhang of deltopectoral crest); 1) flat to convex surface at origin of bracialis (reduced to absent lateral overhang of deltopectoral crest).

119. Humerus (medial view), deltopectoral crest, deltoid tuberosity: 0) present (prominent); 1) absent (reduced).

120. Humerus (posterior view), fossa for M. triceps brachii: 0) absent; 1) present.

121. Humerus, lateral epicondylar crest: 0) well developed (border in posterior view convex and projecting postero-laterally); 1) absent or poorly developed (border in posterior view straight to weakly convex and weakly projecting postero-laterally); Modified [11, 12].

122. Humerus, entepicondylar foramen: 0) present; 1) absent; [12, 22].

123. Humerus, medial epicondyle, posterior and anterior view: 0) projecting medially, with rounded proximal and distal borders (distal end separated from troclear); 1) projecting medially, with oblique proximal and distal borders; 2) does not project medially, lacking proximal and distal borders.

124. Humerus, lesser tubercle and head: 0) equal in height or tubercle insignificantly higher than head, 1) tubercle much higher than head; [7, 14].

125. Humerus, greater tubercle: 0) level (or close to) or lower than head; 1) much higher than head.

126. Humerus, bicipital ridge (transverse bar): 0) absent; 1) present.

127. Humerus, bicipital groove: 0) greater and lesser tubercles do not enclose the bicipital groove antero-medially; 1) greater and lesser tubercles enclose the bicipital groove anteromedially.

128. Humerus, distal portion of shaft: 0) more developed transversely relative to proximal portion; 1) less developed transversely relative to proximal portion; [7, 17].

129. Radius, location of radial tuberosity: 0) on medial side; 1) on posteromedial side; [12].

130. Radius, deep groove for extensor digitorum communis tendon: 0) absent; 1) present; [12].

131. Ulna, articular facet for cuneiform (=pyramidal): 0) transversely oriented; 1) slightly inclined anteroposteriorly; 2) nearly perpendicular relative to main axis of ulna; [7, 17].

132. Ulna, anconeal tuberosity: 0) well developed and posteriorly positioned on olecranon; 1) well developed and anteriorly positioned on olecranon; 2) poorly developed; [7, 25].

133. Trapezium: styloid process (forming medially the scapholunar facet and laterally the facet for the carpale II): 0) long; 1) short; [12].

134. Cuneiform, distally projecting ledge (palmar process): 0) absent; 1) present; [12, 22].

135. Metapodials, head: 0) keeled with trochleated phalangeal articulations; 1) smooth, with phalanges flat, articulations hingelike; Modified [11, 12].

136. Metacarpal I and metacarpal II: 0) approximately the same length; 1) metacarpal 1 longer than metacarpal II; [7, 15, 19].

137. Manus, digit V, intermediate phalanx: 0) unreduced; 1) strongly reduced; [12, 22].

138. Manus, phalanges: 0) rounded; 1) flattened; [7, 15].

139. Manus claws: 0) large; 1) small; Modified [12, 22].

140. Sacrum, sacral wings (lateral view): 0) orientated antero-posteriorly (diagonal); 1) orientated dorsal-ventrally (vertical); 2) oriented dorsal-ventrally (vertical), with lateral edges flaring anteriorly.

141. Innominate, ilium: 0) shallow gluteal fossa and weakly everted wing; 1) deep gluteal fossa and strongly everted wing; [12].

142. Innominate, location of anterodorsal iliac spine: 0) dorsal to the anteroventral iliac spine; 1) posterodorsal to the anteroventral iliac spine; [12].

143. Innominate, posteroventral iliac spine (= iliac tuberosity, [26]): 0) large and strongly protruding; 1) small or absent; [12].

144. Innominate, proportions of postacetabular region (width/length): 0) long and narrow (ratio less than or equal to 0.5); 1) short and wide (ratio greater than 0.6); [12, 22].

145. Innominate, ishial spine: 0) unenlarged; 1) large; [11, 24].

146. Femur, collo-diaphyseal angle: 0) high, head oriented more medially than proximally; 1) low, head oriented more proximally than medially; [12].

147. Femur, epicondyle crest: 0) inconspicuous or short; 1) long (reaching at least the mid-length of the diaphysis); [12].

148. Femur, proportions (length/mid-shaft width): 0) long and narrow (ratio greater than 2.6); 1) short and wide (ratio less than 2.6); Modified [12].

149. Femur, condyles in posterior view: 0) different in size; 1) similar in size Modified [7, 14].

150. Femur epiphyses: 0) distal epiphysis one-fourth to one-fifth wider than proximal; 1) widths of proximal and distal epiphyses about equal; 2) proximal epiphyses wider than distal (medial) one; [7, 14].

151. Femur orientated sitting on condyles, head and greater trochanter: 0) same level or head higher; 1) greater trochanter slightly higher than head; Modified [7, 13, 16, 22].

152. Femur, neck: 0) long, slender; 1) short, wide; [7, 14].

153. Femur, trochlea: 0) slightly higher than wide; 1) slightly wider than high; [7].

154. Femur, trochanteric fossa: 0) relatively deep; 1) reduced or absent; [7, 11].

155. Tibia and fibula: 0) not fused; 1) fused proximally; [7, 11].

156. Tibia, posterotibial fossa: 0) shallow; 1) deep; [7, 19].

157. Astragalus, calcaneal process: 0) absent; 1) poorly developed; 2) well developed (ordered); Modified [11, 12].

158. Astragalus, facet for articulation with tibia: 0) not elevated; 1) elevated and forming a calcaneal process; [7, 17].

159. Sustentacular facet of the astragalus: 0) oval-shaped and narrowed at contact with cuboid facet; 1) long (at least twice longer than wide), slender and strongly bent medially; 2) short and tongue-like with no narrowing at contact with cuboid facet; [12].

160. Calcaneum, fibular portion of ectal facet: 0) absent or very reduced (less than 50% of astragular portion); 1) well developed; Modified [12].

161. Calcaneum, length: 0) slightly longer than astragalus; 1) approximately same length as astragalus; 2) shorter than astragalus; [7, 17, 25].

162. Calcaneum, ectal facet: 0) forms an oblique angle with the main axis of the bone; 1) forms a right angle with main axis of bone; Modified [7, 27].

163. Cuboid, contact between posterolateral process and metatarsal V: 0) present; 1) reduced-absent; [7, 17, 25].

164. Metatarsal I, articular surface for metatarsal II (plantar face): 0) oriented laterally; 1) oriented proximal-laterally; 2) inconspicuous; Modified [12].

165. Metatarsal III, length: 0) less than 50% shorter than metatarsal I; 1) approximately 50% shorter (or more) than metatarsal I; Modified [12, 22].

166. Metatarsal V, articular surface (medial view): 0) greater than 90 degrees from proximal dorsal plane; 1) less than 90 degrees from proximal-dorsal plane.

167. Hindflipper, ungual phalanges: 0) unreduced; 1) reduced; [7, 11].

168. Pes claws: 0) large; 1) small; Modified [12, 22].

5. Character coding

Supplemental Table 5. Morphological character coding for each operational taxonomic unit. "?" denotes unknown character coding. "-" denotes non-applicable character coding.

Hadrokirus martini	111101100000211100001111111011?01002113011101010111010100011000?011001000210000132--??????01000011?10?1?101???102111?0?1????
Acrophoca longirostris	10011??0??012?11101021000?010100010020020111110121010111000110000011001000211000132--12011101000011011?1?100011110?000001120010101110011?0?100111011110101102121001101??
Piscophoca pacifica	111101?0?00021110??011000?0101001010[1,2]0131111010100110101000110000011001000210000132--?????10101001101??1?010?110100001011110010101?10011?0??00111?????????00211100?1??1?
Homiphoca capensis	11010??1?1002?11001111010???0??00110?0120111010101010111001001100001001000110000132--10011101000011 0???????????????000001010010100????0??????????0?0010110?12111101111??
Monotherium? wymani	??0???0?0?10010??2110??131--10011?010000??
Australophoca changorum	?? 0001011[1,2]00001???0?????????????0?????0?0??211000??????
Sarcodectes magnus	110111?1?0002?1000112011 0?????????????230111010?11010101???0100100?10010??1100?01310-1001100101001??????????0??????00??0111?0?????????1??1???????????????????????????????
Pliophoca etrusca	1101101000?0??100001101111??11?0????0?3?111?10?????0???00?1???0???100??????????????11?00???1?000???1???????? ??????0010011100?010?200?11?1???????01?100010??212?00111?1?
Arctocephalus sp.	0100011000101000000010001101001000000101000010010210000000100000000000-00-0000-010100--0-000?000--0000000010100000011010000000100000000000111100010000011001111000000020001
Cystrophora cristata	1001111000101010--0000000001111-0000101110100201200110010001101000010010011101001200010111021001111010110000101010011100001100001000011100011110100000110011212000100110
Erignathus barbatus	11001110011021100000100000010100011000010011010120011001010011110001001000100000013000100110110001111100100001010100110000011000010000100000010000100002100011202000010100

Halichoerus grypus	110001100000111000-0[0,1]000000101100110100100100102200110010101100000010010011001001201010?110210001110100100001010100110000011000110000101010211001000020010112110000010100
Pusa sibirica	100001100000211010002000000111100010200100110102220110010101101000010110011001011201010021?2100111101001000010100001100100110010100001000101110010000210100121101000010 0
Histriophoca fasciata	11000110000020100000[0,1]00000011110130020020011010120011001010110100001011001120101120001 0?11?2100111101101000010101001100000110000100001 00?0021100100002111001211020010100
Pagophilus groenlandicus	100001100010211000001000000101100010000200110102200110010101101000010110011201011200010011021001111010110000101010011001001100001000010001011100100002111001211010010100
Phoca vitulina	110001100000211000001000100111100010200200100102200100010101101000010110011001011201010011021001111110010000111010011000001100101000010101021100100002011011211010000100
Pusa hispida	100001100000211010001000000101101010001100110102200100010101101000010110011001001201010021021001111010010000101010011000001100101000010001021100100002101011211000000100
Phoca largha	1000011000002110000010000001011010101002001101010001000101011010000101100110010012010 1??11?21000111010010??
Pusa caspica	11001110000021100000100000001?11000??2?01001101022001000101011010000101100110010112010 10?110210011110100100001010100110000011001010000100000211001000011010112110100001 00
Mirounga leonina	1101011000101010--0000000001211-12000010-111010120011101000011001001001010002000 1300-10101?0110001101011 0??????????0????11?1???10011101101??????????????????????????????
Mirounga angustirostris	100101100010111---0000000001211-02000020-1110101200111010000110010010010100020001300-1011100110001101011001001101000011011210001001 21011?1?1011111110101111 0212020?1111?
Monachus monachus	110110100010211000011011111101100200000301110101000101010001100000010011002100001 10211?001?110100110110100110110101001001110010100210011011100111010110010102121 00111111
Neomonachus tropicalis	1101001000101110000121110121011001002002011101010101010100011000000100110021000011000 1000001100001101001001101101010010011100001002010110 1?1001110101100111021211011011?
Neomonachus schauinslandi	110100100010211000012001110101100100201301100102010101010001100000010011002100001100001000011010011010010001011010000110111000010000001111110011100011001100212110111111
Lobodon carcinophaga	11010110101021111100210000010110110100121110110121000011000100000000100100020000 0132--1201100101001101001000001111010111011210001102010111111001110100001111121212 0111111

Hydrurga leptonyx	10010110000021111001110001010111100020031111110101010011000010000011001000200000132--11111?0100001101101011001111010110011110001102010111111001110100201111212120111111
Ommatophoca rossi	11110110101020110000100000010110010000111111-01210000110011000000111010000000000131--11011001100111011010010001110101110112100011021101111110011101101011110212120111111
Leptonychotes weddelli	1101011010002111-00000000101011111002013011111020000001100010000001100100020000 0132--12011001000011110010110001110101101111100011010101111110111101001011110212120111111
Potamotherium valletoni	000011?000000-00001?-0-10?-000100??00-000000?10120000?001001100??000000000???000100?-???000000--110100?0?000?10000000?0?00000?00100000000?000011000001000000000000100??0
Enaliarctos emlongi	1100110000100?00-01000010101000-00000-01000001022000000010000000000000-0000--00000--?????00000000000000???0000101?001????????????
Allodesmus kernensis	110000?0?0001100-???00010??0011000102?10010001022000001100000000000-0000000-000-00--?????0000--1010???0?011001?0??011101100000000000010?1?2011?000001001000000000?01??
Devinophoca claytoni	11001??0??100110?0?????10???????????????201010?0220?101??0001?0100001001000???000120?-??????010000110???
"*Leptophoca proxima*" CMM-V-2021	1100111000102?1??000??????01111200100?1300110102210111010101??00010100100011100011000??????0100011?1??1?0000???
Nanophoca vitulinoides	???1??????1010000100001100 0???????2111??0000200001??????0??????
Odobenus rosmarus	100????????01?1?--00000101?0000-?210?0-000001001200?0000001000000000000000000?000000--00000?000--00100000010000010000001101100000000100111111001111000020011101000001 20100

6. Parsimony Analysis Data

Supplemental Table 6. Synapomorphies of clades resolved from the equal weights parsimony phylogenetic analysis. Unequivocal synapomorphies for the tree are indicated in bold.

Clade	Character Synapomorphies
Phocidae	15, **52**, **54**, **68**, **71**, **82**, **93**
Phocinae	35, 53, **58**
Monachinae	**4**, **34**, 82, 121, 122, 128, 131, 135, **147**, **165**, **167**, 168
Clade 1	119
Lobodontini-Miroungini	124, 152, **154**, 161
Miroungini	13, 21, 29, 34, 40, 53, 60, 62, **65**, **73**, 77, 88, 94, 103
Lobodontini	**9**, 16, 45, **52**, 54, 61, **83**, 87, **112**, 115, **117**, 137, 160
Clade 2	49, 76, 160
Peruvian-South African clade	16, 22, **67**, 83, **91**, 132, 138, 159
Peruvian-South African clade + *Sarcodectes magnus–Monotherium? wymani*	49, 76, 160
Sarcodectes magnus–Monotherium? wymani	60, 83
Monachini	**6**, 72, 82, 89, 92

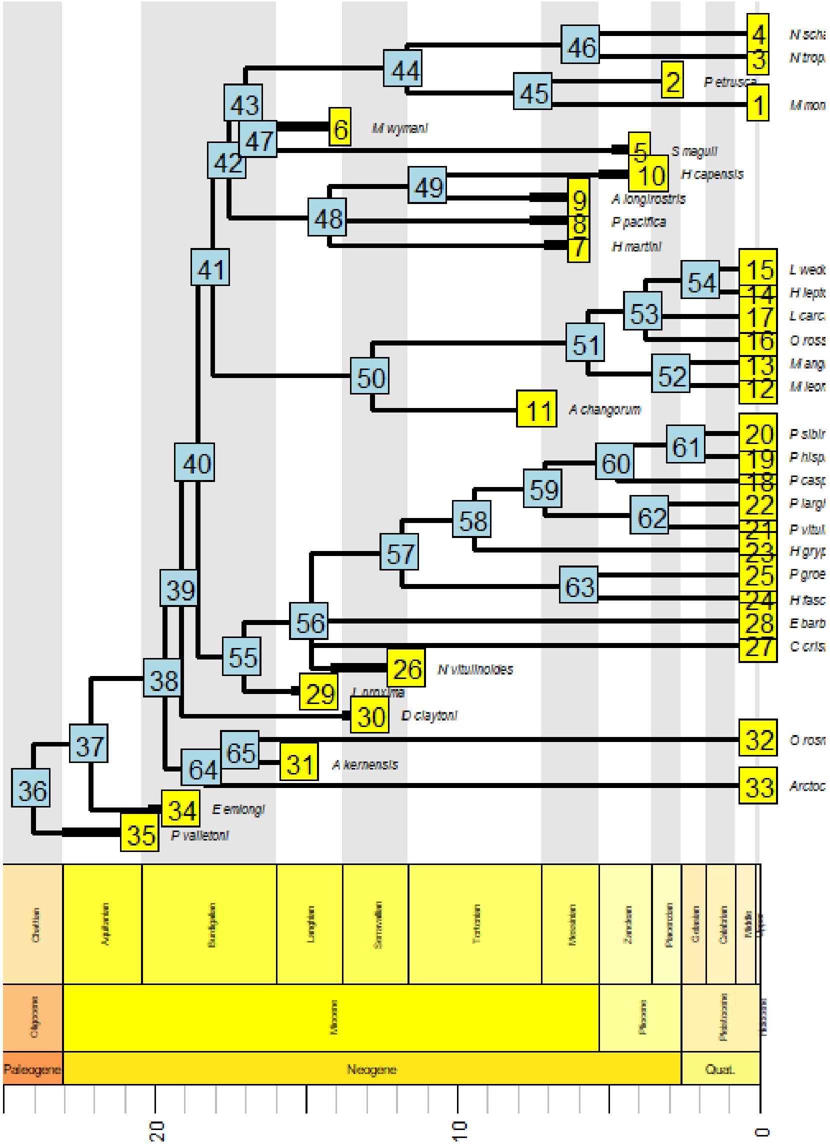

Supplemental Figure 6. Equal weights parsimony tree (combined 4 most parsimonious tree), with tip and node labels. Constructed using R package "strap".

Supplemental Table 7 Tip labels for Supplemental Figure 4.

EW Tip	Taxon
1	*Monachus monachus*
2	*Pliophoca etrusca*
3	*Neomonachus tropicalis*
4	*Neomonachus schauinslandi*
5	*Sarcodectes magnus*
6	*Monotherium? wymani*
7	*Hadrokirus martini*
8	*Piscophoca pacifica*
9	*Acrophoca longirostris*
10	*Homiphoca capensis*
11	*Australophoca changorum*

12	*Mirounga leonina*
13	*Mirounga angustirostris*
14	*Hydrurga leptonyx*
15	*Leptonychotes weddellii*
16	*Ommatophoca rossii*
17	*Lobodon carcinophaga*
18	*Pusa caspica*
19	*Pusa hispida*
20	*Pusa sibirica*
21	*Phoca vitulina*
22	*Phoca largha*
23	*Halichoerus grypus*
24	*Histriophoca fasciata*
25	*Pagophilus groenlandica*
26	*Nanophoca vitulinoides*
27	*Cystophora cristata*
28	*Erignathus barbatus*
29	*"Leptophoca proxima"*
30	*Devinophoca claytoni*
31	*Allodesmus kernensis*
32	*Odobenus rosmarus*
33	*Arctocephalus* sp.
34	*Enaliarctos emlongi*
35	*Potamotherium valletoni*

Supplemental Table 8. Node ages calculated using the "strap" package in R. NA = Non-applicable.

**Node**	**EW Length (Millions of years)**
34	**24.03**
35	**22.52**
36	**19.42**
37	**17.84**
38	**14.23**
39	**12.2**
40	**10.16**
41	**8.13**
42	**6.1**
43	**4.07**
44	**2.03**
45	**5.09**
46	**15.62**
47	**11.48**
48	**1.6**
49	**7.33**

50	**14.98**
51	**7.49**
52	**14.33**
53	**13.69**
54	**11.12**
55	**9.18**
56	**9.18**
57	**4.83**
58	**2.42**
59	**2.42**
60	**16.17**
61	**16.09**
62	**NA**
63	**NA**
64	**NA**
65	**NA**

Supplemental Table 9. Stratigraphic range data for each taxon, with references.

Taxon	Upper limit	Lower limit	Reference
Potamotherium valletoni	23.03	20.44	[28]
Enaliarctos emlongi	20.2	19.1	[29]
Allodesmus kernensis	15.85	15.15	[29]
Devinophoca claytoni	13.82	12.829	[30, 31]
"Leptophoca proxima" (CMM-V-2021)	15.5	14.5	[32]
Nanophoca vitulinoides	14.2	11.63	[5]
Sarcodectes magnus	4.9	3.9	[32]
Pliophoca etrusca	3.19	2.82	[7]
Homiphoca capensis	5.33	3.6	[33, 34]
Monotherium? wymani	15.97	13.8	[35]
Acrophoca longirostris	7.6	5.9	[9, 32]
Piscophoca pacifica	7.6	5.9	[9, 32]
Hadrokirus martini	7.1	5.9	[32]
Australophoca changorum	7.6	7.3	[9]

7. References

1. Koretsky IA, Ray CE. 2008 Phocidae of the Pliocene of eastern USA. *Virginia Mus. Nat. His. Spec. Pub*. **14**: 81–140.
2. Churchill M, Clementz MT, Kohno N. 2014 Predictive equations for the estimation of body size in seals and sea lions (Carnivora: Pinnipedia). *J. Anat*. **225:** 232-245.
3. Piérard J, Bisaillon A. 1978 Osteology of the Ross Seal *Ommatophoca rossi* Gray, 1844. *Antarcr. Res. Ser.* **31:** 1–24.
4. Pierard J. 1971 Osteology and myology of the Weddell seal *Leptonychotes weddelli* (Lesson, 1826). *Antarct. Res. Ser*. **18:** 53–108.
5. Dewaele L, Amson E, Lambert O, Louwye S. 2017 Reappraisal of the extinct seal "Phoca" vitulinoides from the Neogene of the North Sea Basin, with bearing on its geological age, phylogenetic affinities, and locomotion. *PeerJ*. **5**: e3316.
6. Dewaele L, Peredo CM, Meyvisch P, Louwye S. 2018 Diversity of late Neogene Monachinae (Carnivora, Phocidae) from the North Atlantic, with the description of two new species. *R. Soc. Open. Sci*. **5**: 172437. (10.1098/rsos.172437)
7. Berta A, Kienle S, Bianucci G, Sorbi S. 2015 A Reevaluation of *Pliophoca etrusca* (Pinnipedia, Phocidae) from the Pliocene of Italy: Phylogenetic and Biogeographic Implications. *J. Vertebr. Paleontol*. **35**: e889144.
8. King J. 1983 *Seals of the world*, London: British Museum (Natural History).
9. Valenzuela-Toro AM, Pyenson ND, Gutstein CS, Suarez ME. 2016 A New Dwarf Seal from the Late Neogene of South America and the Evolution of Pinnipeds in the Southern Hemisphere. *Pap. Palaeontol*. **2**: 101–115. (10.1002/spp2.1033)
10. Churchill M, Clementz MT, Kohno N. 2015 Cope's rule and the evolution of body size in Pinnipedimorpha (Mammalia: Carnivora). *Evolution*. **69**: 201–215.
11. Berta A, Wyss A. 1994 Pinniped phylogeny. *Proc. San Diego Soc. Nat. Hist*. **29:** 33–56.
12. Amson E, de Muizon C. 2014 A new durophagous phocid (Mammalia: Carnivora) from the late Neogene of Peru and considerations on monachine seals phylogeny J. Syst. Palaeontol. **12:** 523–548.
13. de Muizon C. 1982 Phocid phylogeny and dispersal. Ann. S. Afr. Mus. **89:** 175–213.
14. Koretsky IA, Grigorescu D. 2002 The fossil monk seal *Pontophoca sarmatica* (Alekseev)(Mammalia: Phocidae: Monachinae) from the Miocene of eastern Europe. *Smithson. Contrib. Paleobiol.* **93:** 113–148.
15. Wyss AR. 1988 On "retrogression" in the evolution of the Phocinae and phylogenetic affinities of the monk seals. *Am. Mus. Novit*. **2924:** 1–38.
16. Bininda-Emonds OR, Russell AP. 1996 A morphological perspective on the phylogenetic relationships of the extand phocid seals (Mammalia: Carnivora: Phocidae). Bonner Zool. Monogr. **41:** 1–256.
17. Sorbi S. 2004 La *Pliophoca etrusca* (Mammalia: Pinnipedia) del Pliocene toscano: nuova descrizione ed inquadramento filogenetico. *Tesi di Laurea Universita di Pisa.* Pisa, Italy.
18. de Muizon C. 1981 Premier signalement de Monachinae (Phocidae, Mammalia) dans le Sahélien (miocène supérieur) d'Oran (Algérie). *Palæovertebrata*.**11 (5):** 182–194.
19. Cozzuol MA. 2001 A "northern" seal from the Miocene of Argentina: implications for phocid phylogeny and biogeography. *J. Vertebr. Paleontol*. **21**: 415–421.
20. Adam P, Berta A. 2002 Evolution of prey capture strategies and diet in the Pinnipedimorpha (Mammalia, Carnivora). *Oryctos*. **4**: 83–107.
21. Wyss AR. 1987 The walrus auditory region and the monophyly of pinnipeds. *Am. Mus. Novit.***2871:** 1–31.
22. King JE. 1966 Relationships of the hooded and elephant seals (genera *Cystophora* and *Mirounga*). *J. Zool*. **148**: 385–398.
23. Nojima T. 1990 A morphological consideration of the relationships of pinnipeds to other carnivorans based on the bony tentorium and bony falx. *Mar. Mam. Sci*. **6**: 54–74.
24. Dewaele L, Lambert O, Louwye S. 2017 On *Prophoca* and *Leptophoca* (Pinnipedia, Phocidae) from the Miocene of the North Atlantic realm: redescription, phylogenetic affinities and paleobiogeographic implications. *PeerJ*. **5**: e3024.
25. de Muizon C. 1981 Les vertébrés fossiles de la Formation Pisco (Pérou). Premiere paretie: deux nouveaux Monachinae (Phocidae, Mammalia) du Pliocene de Sud-Sacaco. *Memoire de l'Institut Francais d'Etudes Andines*. **50:** 1–88.
26. Miller M, Christensen G, Evans H. 1964 Anatomy of the dog. *Anatomy of the dog.* St. Louis, Missouri: Elsevier.
27. Sorbi S. 2004 Primo ritrovamento di Pliophoca (Mammalia: Pinnipedia) nel Pleistocene toscano: descrizione e comparazione. *Tesi di Laurea Specialistica, Universita di Pisa.* Pisa: Italy.
28. Savage RJG. 1957 The anatomy of *Potamotherium* an Oligocene lutrine. *Proc. Zool. Soc. Lond.* **129:** 151–244.
29. Boessenecker RW, Churchill M. 2018 The last of the desmatophocid seals: a new species of Allodesmus from the upper Miocene of Washington, USA, and a revision of the taxonomy of Desmatophocidae. *Zoolo. J Linnean Soc*. **184**: 211–235.
30. Sabol M, Holec P. 2002 Temporal and spatial distribution of Miocene mammals in the Western Carpathians (Slovakia). *Geol. Carpath*. **53**: 269–279.
31. Hohenegger J, Ćorić S, Wagreich M. 2014 Timing of the middle miocene Badenian stage of the central Paratethys. *Geol. Carpath.* **65**: 55–66.
32. Marx FG, Fordyce RE. 2015 Baleen boom and bust: a synthesis of mysticete phylogeny, diversity and disparity. *R. Soc. Open Sci*. **2**: 140434.
33. Hendey QB, Repenning C. 1972 A Pliocene phocid from

South Africa. *Ann. S. Afr. Mus*. **59 (4):** 71–98.
34. Avery G, Klein RG. 2011 Review of fossil phocid and otariid seals from the southern and western coasts of South Africa. *Trans. R. Soc. S. Afr*. **66**: 14–24.
35. Dewaele L, Lambert O, Louwye S. 2018 A critical revision of the fossil record, stratigraphy and diversity of the Neogene seal genus Monotherium (Carnivora, Phocidae). *R. Soc. Open Sci*. **5**: 171669.
